# Commentary: AVneo—Impressive early results; however, long-term durability remains unknown

**DOI:** 10.1016/j.xjon.2021.09.012

**Published:** 2021-09-17

**Authors:** Scott C. DeRoo, Gabriel S. Aldea

**Affiliations:** Division of Cardiac Surgery, University of Washington, Seattle, Wash

**Figure:**
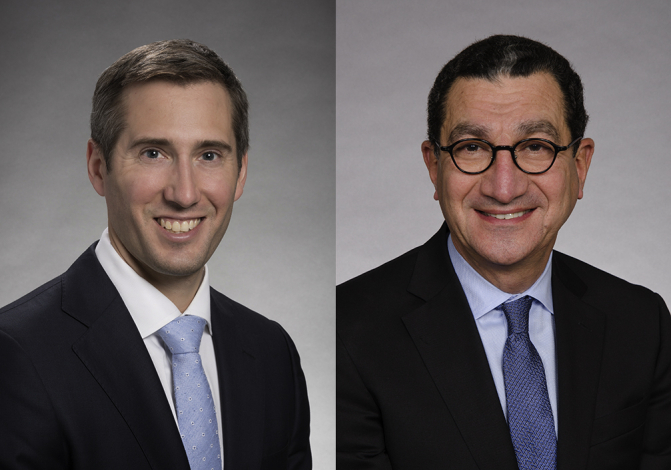
Scott C. DeRoo, MD, and Gabriel S. Aldea, MD

**Central Message**

AVNeo is a unique alternative to traditional options for aortic valve replacement; however, long-term outcomes remain uncertain.

See Article page 193.

In this issue of *JTCVS Open*, Khatchatourov and colleagues^1^ present an impressive series of 70 patients who underwent aortic valve replacement via reconstruction of a new valve with native or xenograft pericardial tissue. This technique, first described by Ozaki and colleagues in 2011, offers an alternative to conventional valve replacement. Consistent with previous results, the authors of the associated study demonstrate excellent hemodynamics of the reconstructed aortic valves with impressive outcomes at 24 months.

Of the 70 study patients, only 2 patients (3%) required reoperation during the 24-month follow-up period. No patient suffered clinically significant permanent stroke, and only 2 patients (3%) required insertion of a permanent pacemaker, an impressive feat. In addition, observed hemodynamics were excellent, with mean transvalvular gradients of 13 ± 7 mm Hg at most recent follow-up. These results compare favorably with commercially available valves and speak to the success of AVneo in experienced hands.

However, although the short-term outcomes reported in this study are to be commended, there remain longer-term questions regarding durability and the ultimate applicability of AVneo. Importantly, it is critical to define which patients stand to derive clinical benefit from AVNeo. The decision to perform this technique must be weighed against the implantation of a modern commercially available stented or stentless valves in older patients, or even a Ross procedure in younger patients. In the associated study, the average age of patients was 62 ± 11 years. While there certainly are concerns regarding bioprosthetic valve durability among younger patients, a well-chosen commercial bioprosthesis in a 62-year-old patient should be expected to last 12+ years. Upon surgical valve failure, many, if not all, of these patients would be well-served for the remainder of their lifetimes with a valve-in-valve transcatheter aortic valve replacement (TAVR) implant, provided the surgically implanted valve has a large enough inner diameter. It is of critical importance, therefore, that index valve choice be done with respect to potential future valve-in-valve TAVR. The ability to place a transcatheter valve in patients who have undergone AVNeo remains largely unknown; however, there are several serious theoretical risks, namely coronary obstruction, that are likely to limit routine TAVR.

Additionally, while younger patients can certainly benefit from avoiding long-term anticoagulation, it is important to remember that consensus guidelines demonstrate a small but persistent survival advantage for patients <55 years of age who undergo mechanical versus biologic aortic valve replacement. Moreover, recent work by El-Hamamsy and colleagues^2^ has demonstrated excellent long-term Ross outcomes in young patients, thus reinvigorating this as an excellent alternative option for younger patients in need of aortic valve replacement.

Most importantly, follow-up for AVNeo remains in its relative infancy. We know that most bioprosthetic valves don't fail until at least 10+ years. Although the 2-year results of AVNeo are quite good, the longest reported follow-up for patients who have undergone this procedure is 5 years.^3^ It will be critically important to evaluate 10+ year outcomes when judging this technique against known commercial options. At present, widespread adoption of this technique remains premature.
